# Lesion Network Mapping of Acute Neurological Deficits and Its Prognostic Value After Ischemic Stroke

**DOI:** 10.1016/j.nicl.2025.103895

**Published:** 2025-10-30

**Authors:** Aslı Akdeniz, Ana Sofía Ríos, Uchralt Temuulen, Jochen B. Fiebach, Kersten Villringer, Huma Fatima Ali, Ahmed Khalil, Ulrike Grittner, Thomas Liman, Matthias Endres, Anna Kufner

**Affiliations:** a Max Planck Institute for Human Cognitive and Brain Sciences, Max Planck School of Cognition, Leipzig, Germany; b Charité – Universitätsmedizin Berlin, corporate member of Freie Universität Berlin and Humboldt-Universität zu Berlin, Klinik für Neurologie mit Experimenteller Neurologie, 12203 Berlin, Germany; c Charité - Universitätsmedizin Berlin, corporate member of Freie Universität Berlin and Humboldt-Universität zu Berlin, Center for Stroke Research Berlin (CSB), 12203 Berlin, Germany; d Department of Neurology, Max Planck Institute for Human Cognitive and Brain Sciences, Leipzig, Germany; e Charité – Universitätsmedizin Berlin, corporate member of Freie Universität Berlin and Humboldt-Universität zu Berlin, Institute of Biometry and Clinical Epidemiology, 10117 Berlin, Germany; f Berlin Institute of Health at Charité – Universitätsmedizin Berlin 10117 Berlin, Germany; g German Center for Cardiovascular Research (Deutsches Zentrum für Herz Kreislauferkrankungen, DZHK), Partner Site Berlin 10117 Berlin, Germany; h German Center for Neurodegenerative Diseases (Deutsches Zentrum für Neurodegenerative Erkrankungen, DZNE), Partner Site Berlin 10117 Berlin, Germany; i International Graduate Program of Medical Neurosciences, at Charité – Universitätsmedizin Berlin 10117 Berlin, Germany; j Einstein Center for Neurosciences Berlin at Charité – Universitätsmedizin Berlin 10117 Berlin, Germany; k German Center for Mental Health (Deutsches Zentrum für Psychische Gesundheit, DZPG), Partner Site Berlin 10117 Berlin, Germany

**Keywords:** Ischemic stroke, Lesion-network mapping, Connectome, Functional outcome, Stroke severity

## Abstract

**Background:**

Predicting functional recovery after ischemic stroke is vital for guiding clinical care. This study investigated whether lesion network mapping (LNM), a technique for modeling symptom-specific brain networks, can improve outcome prediction of functional recovery up to one-year post-stroke.

**Methods:**

We pooled data from two prospective stroke cohorts (1000Plus and PROSCIS-B; N = 565). Seven NIHSS-derived symptom networks were generated using LNM based on NIHSS sub-scores on admission (i.e., consciousness, language, motor, sensory, vision, neglect and ataxia). Lesion masks derived from MRI (within 7 days) were intersected with each symptom network to calculate individual network damage scores. Functional outcome was defined by the modified Rankin Scale (mRS) at 3 months (1000Plus) or 12 months (PROSCIS-B). Ordinal logistic regression models were performed to evaluate additional predictive value of LNM: Model 1 included age, lesion volume, and presence of selected neurological deficits; Model 2 included age, lesion volume, and NIHSS-derived network damage scores. Models were compared using pseudo-R^2^ and AIC.

**Results:**

Patients had a mean age of 68 years and a median NIHSS of 3 (IQR 1–5). LNM revealed distinct, symptom-specific networks, with corresponding damage scores that were higher in patients exhibiting the respective deficits compared to those without. However, inclusion of these scores did not enhance the predictive accuracy of functional outcomes beyond that achieved with clinical variables alone (Model 1 vs. Model 2: pseudo-R^2^: 0.0468 vs. 0.0159; AIC:1730.598 vs. 1769.222).

**Conclusions:**

LNM-derived scores reflected symptom topography but did not enhance prediction of functional recovery. While promising as a mechanistic tool, the clinical utility of LNM-based damage metrics for prognostication remains limited and requires further validation.

## Highlights

• Lesion network mapping (LNM) modeled NIHSS-derived neurological deficits in ischemic stroke.
• NIHSS-derived symptom networks were identified for seven clinical categories.
• NIHSS-derived network damage scores did not outperform clinical variables for outcomes.
• NIHSS admission score and age were the most robust predictors of post-stroke recovery.
• These findings suggest limited clinical utility of LNM in terms of outcome prediction of functional recovery after ischemic stroke.

## Graphical abstract

**Figure:**
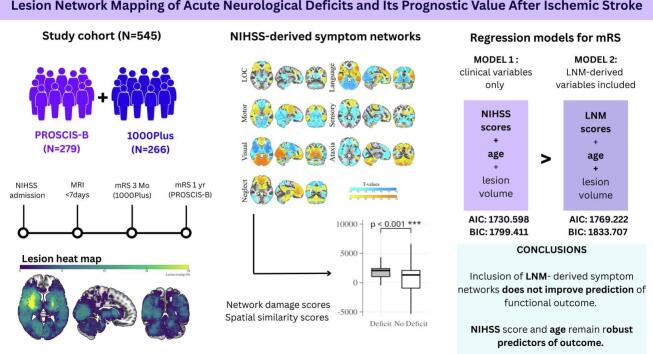

## 1 Introduction

Ischemic stroke, which accounts for nearly 70 % of all strokes worldwide, is a leading cause of long-term disability and reduced quality of life (Gbd, 2021). Accurately predicting stroke outcomes and functional dependency is critical for optimizing recovery strategies and tailoring rehabilitation approaches (Goyal et al., 2021). Improved estimates of clinical outcomes could enable more personalized stroke management, allowing healthcare providers to refine treatment plans while providing better guidance to patients and their families. Previous research has established age, stroke severity, and initial lesion volume as robust, independent predictors of functional outcome after ischemic stroke (Thijs, 2000, Sperber et al., 2023, Rost, 2016, Adams, 1999). However, the variability in recovery between individuals remains poorly understood, with some patients recovering neurological function while others experience permanent impairment (Grefkes and Fink, 2020). This highlights the need for further research into the complex factors that influence recovery outcomes.

Functional neuroimaging studies have advanced our understanding of the neural mechanisms underlying recovery after stroke, providing insights into how to better target and engage neural networks during rehabilitation (Grefkes and Fink, 2020, Cramer, 2008, Chollet, 1991). A key concept, diaschisis − network effects that occur distant from the lesion − plays a central role in both the initial deficits and their recovery over time (Carrera and Tononi, 2014). In other words, an assessment of individual differences in terms of lesion effects on selected symptom networks might aid in predicting stroke recovery.

Lesion network mapping (LNM), which uses normative resting-state functional connectivity data, has emerged as a powerful tool for identifying functional network perturbations caused by focal lesions (Fox, 2018, Zarifkar et al., 2023). A recent study of > 7500 patients showed that not only were lesions that disrupted symptom-related networks, particularly those associated with sensory deficits and dysarthria, predictive of poorer functional recovery at three months, but also that assessment of functional symptom networks of acute neurological deficits improved the accuracy of outcome prediction compared to models that included clinical stroke parameters alone (Ding, 2023). While some studies have found that the integration of structural and functional network data into predictive models has improved the accuracy of outcome prediction in certain domains (e.g., motor and language outcomes) (Ktena et al., 2019, Bowren, 2022) other studies found no added benefit of LNM for predicting cognitive outcomes (Salvalaggio et al., 2020) highlighting that the clinical utility of LNM remains debated (Salvalaggio et al., 2021, Salvalaggio et al., 2021).

In this study, we applied LNM in a pooled analysis of two independent, prospective stroke cohorts to identify distinct functional symptom networks associated with seven specific neurological deficits as measured by the National Institute of Health Stroke Scale (NIHSS). We analyzed seven categories of acute neurological deficit separately, including deficits in level of consciousness (LOC), vision, motor function, sensory function, language, ataxia and neglect. Each category was characterized by an individual NIHSS component score. Our primary aim was to determine whether patient-specific disruption to these NIHSS-derived symptom networks caused by the ischemic lesion (referred to as network damage scores) improved prediction of functional recovery outcomes after stroke as measured by the modified Rankin Scale (mRS).

## 2 Materials and methods

### 2.1 Subjects

We included patients from two independent single-centre prospective studies: the PROSCIS-B (NCT01363856 (Liman, 2013) and 1000Plus (NCT00715533 (Hotter, 2009) studies. The PROSCIS-B study, conducted at the Center for Stroke Research Berlin, Charité University Hospital, enrolled patients aged 18 or older with a first acute stroke occurring within seven days prior to inclusion. Follow-up assessments, including modified Rankin Scale (mRS) scores, were conducted one year after stroke. The 1000Plus study is a prospective, single-centre, observational study conducted at the Benjamin Franklin Campus of Charité University Hospital (EA4/026/08). The study recruited patients aged 18 years or older who were admitted to the emergency department with a clinical diagnosis of acute cerebrovascular event within the previous 24 h and who met general eligibility criteria for magnetic resonance imaging (MRI). Follow-up assessments of functional dependence, as assessed by mRS, were performed three months after stroke. Full details of the PROSCIS-B and 1000Plus study design and protocol are available in the previously published study protocols (Liman, 2013, Hotter, 2009).

For this analysis, patients from both studies were included if they met the following criteria: (1) they underwent at least one MRI within seven days after the event, (2) an acute ischemic lesion was detectable on the MRI, and (3) NIHSS scores, including sub-scores, were available at admission, as well as assessment of functional outcome via the mRS score at either three months or one year post-stroke. All patients provided written informed consent. Both trials received ethical approval from the Berlin Ethics Committee and complied with the ethical principles outlined in the Declaration of Helsinki (PROSCIS: EA1/218/09 and 1000Plus: EA4/026/08).

### 2.2 Neurological deficit and functional outcome assessment

Stroke severity was assessed on admission to the stroke unit by an attending physician certified in the use of the NIHSS. Stroke etiology was determined and categorized based on the TOAST (Trial of Org 10,172 in Acute Stroke Treatment) classification criteria (Adams, et al., 1993). Functional outcomes were assessed via the mRS at 3 months post-stroke for the 1000Plus study population and at 12 months post-stroke for the PROSCIS-B cohort. Admission NIHSS scores were categorized into seven different neurological deficit categories based on the following definitions: level of consciousness (LOC: 1a, 1b, 1c), language deficit (9, 10), motor deficit (4, 5a, 5b, 6a, 6b), sensory deficit (8), visual deficit (2, 3), ataxia (7) and neglect (11). This grouping approach was used to reflect clinically meaningful domains of neurological function and to enable domain-specific analyses of stroke-related impairments. For each domain, patients were classified as having a deficit (“yes”) if any of the corresponding NIHSS items had a score greater than zero; otherwise, they were classified as having no deficit (“no”). For the LNM analyses performed with FSL Randomise, these classifications were binarized (0 = no deficit, 1 = deficit). A detailed description of the questions and examinations included in each category of the NIHSS is given in Table 1.

**Table 1:** **Categorization of Acute Neurological Deficits Based on NIHSS Sub-Scores.** This table outlines the grouping of NIHSS (National Institutes of Health Stroke Scale) components into seven categories of acute neurological deficits.

**Category**	**Score-based grouping**	**Questions / Commands**
Level of consciousness	1a + 1b + 1c	**1a (Alertness):** Assess the overall alertness and responsiveness of the patient. **1b (Questions):** What is the month? How old are you? **1c (Commands):** Ask the patient to open and close their eyes, and then to grip and release the non-paretic hand.
Visual deficit	2 + 3	**2 (Gaze):** Assess horizontal eye movements by asking the patient to follow a moving object with their eyes. **3 (Visual Fields):** Test for visual field loss by asking the patient to identify the number of fingers being held up in each quadrant of their visual field.
Motor deficit	4 + 5 a + 5 b + 6 a + 6 b	**4 (Facial Palsy):** Ask the patient to show their teeth or raise their eyebrows and close their eyes tightly. **5a (Motor Arm Left):** Ask the patient to extend the left arm 90 degrees if sitting or 45 degrees if lying down. Hold for 10 s. **5b (Motor Arm Right):** Ask the patient to extend the right arm 90 degrees if sitting or 45 degrees if lying down. Hold for 10 s. **6a (Motor Leg Left):** Ask the patient to raise the left leg to 30 degrees and hold for 5 s. **6b (Motor Leg Right):** Ask the patient to raise the right leg to 30 degrees and hold for 5 s
Ataxia	7	**7 (Limb Ataxia):** Check for coordination in the arms and legs by performing the finger-nose-finger and heel-shin tests.
Sensory deficit	8	**8 (Sensory):** Test for sensation in the arms, legs, trunk, and face using pinpricks or light touch.
Language deficit	9 + 10	**9 (Aphasia):** Assess spontaneous speech, comprehension, and the ability to name objects and describe a picture. **10 (Dysarthria):** Ask the patient to read or repeat a list of words.
Neglect	11	**11 (Extinction and Inattention):** Check for the patient's ability to pay attention to both sides of the body and to stimuli presented to both sides simultaneously.

### 2.3 Imaging

All patients underwent a standardized imaging protocol on a 3 Tesla MRI scanner (Tim Trio; Siemens, Erlangen, Germany), including diffusion-weighted imaging (DWI; TE = 93.1  ms, TR = 7600  ms, FOV = 230  mm, matrix = 192 × 192, 2.5-mm section thickness with no interslice gap) and fluid-attenuated inversion recovery (FLAIR). Acute ischemic lesions were identified by radiology or neurology trainees under the supervision of a consultant radiologist. Lesions were manually delineated on all visible slices of diffusion-weighted imaging (DWI) scans using MRIcron software (https://www.nitrc.org/projects/mricron) by trained physicians (A.S.R., H.F.A., and A.Ku). The process was overseen by at least two independent expert neurologists and/or radiologists (A.Kh and K.V.), who were blinded to all clinical information. The resulting lesion masks were co-registered to skull-stripped b0 images using FSL’s Brain Extraction Tool (BET) and subsequently normalized to Montreal Neurological Institute (MNI152) standard space with a resolution of 1 × 1 × 1 mm. The normalization workflow used linear and non-linear registration steps after lesion masking, implemented using Advanced Normalization Tools in Python (ANTsPy) (Tustison et al., 2021).

### 2.4 Lesion network mapping

Connectivity profiles for each patient's lesion mask were determined using the Lead-DBS Mapper (Neudorfer, 2023). This approach estimates functional connectivity using a normative connectome constructed from resting-state fMRI data of 1000 healthy individuals (Fox, 2018), as described in prior studies (Yeo et al., 2011). Specifically, the mean BOLD signal within each lesion mask was correlated with all other brain voxels across the normative dataset, resulting in a full-brain connectivity map with identical dimensions across all patients. The resulting maps of Pearson correlation coefficients were subsequently transformed using Fisher’s z-transformation.

LNM analyses were performed on the combined study population of 565 patients from the 1000Plus and PROSCIS-B studies. From the NIHSS, we derived seven LNM-based networks corresponding to core deficit domains (consciousness, language, motor, sensory, visual, ataxia, neglect). For simplicity, we refer to these collectively as NIHSS-derived symptom networks. Each NIHSS sub-score category score was binarized per patient (0 = no deficit, 1 = deficit) for the LNM analyses. Network analysis was performed independently for each of the seven NIHSS symptom categories as follows: using the FSL’s Randomise tool, a two-sample permutation *t*-test was performed with two contrasts, +1: to identify regions associated with lesions leading to a deficit within the symptom category, and −1: to identify regions associated with lesions without a deficit in that category. All analyses were adjusted for lesion volume and NIHSS sum score to control for overall stroke severity and lesion burden. Both covariates were demeaned prior to inclusion in the design matrix. Lesion side (left vs. right) was not taken into account in group-level analyses. This analysis involved 5000 permutations. To account for multiple comparisons, Family-Wise Error (FWE) correction was applied, followed by Threshold-Free Cluster Enhancement (TFCE) to identify cluster-forming voxels within the connected regions (Smith and Nichols, 2009). The final maps represent 1 minus the P-value of the association between NIHSS sub-scores and connectivity profiles, with a significance threshold set at 0.95. Voxels within the identified network were overlaid on several structural atlases, including the Harvard-Oxford Cortical and Subcortical Structural Atlas (Desikan, 2006) and the FNIRT-normalized Cerebellar Atlas in MNI152 space (Diedrichsen et al., 2009), to determine connected anatomical regions.

### 2.5 Network damage scores

For each of the seven categories of neurological deficits, a network damage score was calculated for each individual. First, NIHSS-derived symptom networks were computed by testing the association between lesion connectivity and each NIHSS subscore using FSL Randomise, while controlling for lesion volume and NIHSS sum score. Significant clusters (p < 0.05, FWE-corrected) from these analyses defined the symptom networks associated with each NIHSS subscore (seven in total). Each subject’s network damage score was calculated by adding the intensity (T values) of those voxels in the respective NIHSS-derived symptom network that overlapped with that subject’s lesion, as previously reported (Padmanabhan, 2019). In other words, for each patient, a LOC-network damage score, a language-network damage score, etc., was calculated. This yielded one continuous score per subject per symptom network, with the observed range spanning approximately − 500 to 10,000, as illustrated in Fig. 3*.* All network damage scores were computed using unthresholded symptom networks, which ensures that there is no bias based on which threshold was applied. To control for lesion size, log-transformed lesion volume was regressed out of the damage score, which was then used in all subsequent analyses. To determine whether network damage scores differed between subjects based on the presence or absence of the respective neurological deficit, the Mann-Whitney *U* test was applied between groups (Padmanabhan, 2019).

As a sensitivity analysis, we additionally calculated patient-specific spatial similarity scores to each NIHSS-derived symptom network, following the approach described by Ríos et al (Ríos et al., 2025). Each patient’s individual lesion connectivity profile was compared to the corresponding group-level NIHSS-derived symptom network for that domain: both images were vectorized voxel-wise, and the across-voxel Pearson correlation coefficient (r) between the subject’s connectivity map and the NIHSS-derived symptom network served as the similarity score. These per-domain scores were z-standardized and entered as predictors in two ordinal regression models of mRS to assess prognostic value: a baseline model with age and log-transformed lesion volume, and an extended model adding the similarity scores.

## 3 Statistical analysis

We performed all statistical analyses using STATA IC v17 (StataCorp, College Station, TX) (StataCorp., 2021). Our primary outcome of interest was functional outcome assessed via the mRS at 3 months (1000Plus cohort) and 12 months (PROSCIS-B cohort) after ischemic stroke. Univariate and multivariable ordinal logistic regression models were used to assess the association between selected clinical and imaging parameters and follow-up mRS scores (ordinal variable, range 0–6). The following known characteristics associated with mRS were included: age, admission NIHSS sum score, and baseline log-transformed lesion volume. In the first model, the presence of selected neurological deficits was included based on predefined categories, i.e., for level of consciousness (LOC), language, motor function, sensation, vision, ataxia, and neglect. For these regression models, clinical NIHSS category scores were entered as continuous variables (sum scores). In a second model, clinical scores based on the presence of neurological deficits were replaced by network damage scores obtained from NIHSS-derived symptom networks. Network damage scores were scaled by a factor of 1000 for better interpretability. To compare the models, we calculated the coefficient of determination (R^2^), Akaike Information Criterion (AIC), and Bayesian Information Criterion (BIC) to assess model fit and performance. To further evaluate model performance, we computed the expected mRS scores from the predicted probabilities and plotted the predicted values against the observed values.

The heatmaps illustrate the spatial distribution of lesions superimposed on a 100 µm Edlow background in MNI space. The colour bar indicates the number of lesions overlapping at the voxel level, with yellow representing the highest overlap and blue the lowest. The map highlights the maximum overlap of 24 lesions, providing a visual representation of lesion clustering and their spatial relationship within the entire study cohort, irrespective of NIHSS scores (N = 545).

2D surface maps of the brain highlighting regions functionally connected to lesion sites in patients who developed selected deficits after stroke—loss of consciousness (LOC), language deficit, motor deficit, sensory deficit, visual impairments, and presence of ataxia and neglect. The maps display T-values to facilitate visualization. Each analysis was performed on the total sample of N = 545, with the number of patients classified as having a specific deficit varying by domain.

## 4 Results

### 4.1 Description of study population

The mean age in the pooled patient population (N = 565) was 68.1 (standard deviation [SD] 12.8), median NIHSS on admission was 3 (interquartile range [IQR] 1–5), and median lesion volume was 4.2 mL (IQR 1.5–––13.8). Median time to MRI scan in days was 1 (IQR 0–––1). These values reflect a population with predominantly mild strokes. Patients from the 1000Plus study were older and exhibited higher rates of arterial hypertension, hyperlipoproteinemia, atrial fibrillation, and more frequently had large artery atherosclerosis as the underlying stroke etiology. While the distribution of affected arterial territories was similar between studies, stroke severity was higher in the 1000Plus study population. Lesion laterality was broadly comparable between cohorts, with similar proportions of right- and left-hemispheric lesions. 1000Plus lesions were more evenly distributed (Right = 99, Left = 92, Bilateral = 75), whereas PROSCIS showed a modestly higher proportion of left-sided lesions (Left = 118, Right = 106, Bilateral = 55). Patients from the 1000Plus study had statistically significantly higher mRS scores at 3-month follow-up compared to patients from the PROSCIS study who were assessed at 1-year post-stroke (median 2, IQR 0–3 vs. median 1, IQR 0–2; p < 0.001). Within the pooled study population, median follow-up mRS was 1 (IQR 0–2). For a detailed description of patient demographics based on the study and within the pooled cohort, refer to Table 2**.**

**Table 2:** Baseline Characteristics and Clinical Features of the Study Cohorts.

	**PROSCIS-B** **n = 279**	**1000Plus** **n = 266**	**Total cohort** **(n = 545)**
Age, mean (±SD)	65.5 (13.7)	70.9 (11.0)	68.1 (12.8)
Sex, female, n (%)	102 (36.6)	98 (36.8)	200 (36.7)
Hypertension, n (%)	173 (62.0)	216 (81.2)	389 (71.4)
Diabetes, n (%)	64 (22.9)	69 (25.9)	133 (24.4)
Hyperlipidemia, n (%)	57 (20.4)	156 (58.6)	213 (39.1)
Atrial fibrillation, n (%)	51 (18.3)	77 (29.1)	128 (23.5)
TOAST Classification (Adams 1993) Large artery atherosclerosis, n (%) Cardioembolic stroke, n (%) Small vessel occlusion, n (%) Other, n (%) Unknown, n (%)	89 (31.9) 63 (22.6) 33 (11.8) 15 (5.4) 79 (28.3)	139 (52.3) 82 (30.8) 22 (8.3) 6 (2.3) 11 (4.1)	228 (41.8) 145 (26.6) 55 (10.1) 21 (3.9) 11 (2.0)
NIHSS on admission, median (IQR) Level of consciousness, n (%) Language deficit, n (%) Motor deficit, n (%) Sensory deficit, n (%) Visual deficit, n (%) Ataxia, n (%) Neglect, n (%)	2 (1–4) 21 (7.5) 134 (48.0) 162 (58.1) 81 (29.0) 46 (16.5) 48 (17.2) 17 (6.1)	4 (2–6) 52 (19.6) 156 (58.7) 193 (72.6) 84 (31.6) 75 (28.2) 51 (19.2) 28 (10.5)	3 (1–5) 73 (13.4) 390 (53.2) 355 (65.1) 165 (30.3) 121 (22.2) 99 (18.2) 45 (8.3)
Vascular territory affected: MCA, n(%) ACA, n(%) PCA, n(%) AchA, n(%) Infratentorial (AICA, SUCA, PICA, pons and mesencephalon), n(%) Thalamus, n(%) Multiple territories, n(%)	127 (45.5) 1 (0.36) 16 (5.7) 21 (7.5) 48 (17.2) 29 (10.4) 36 (12.9)	131 (49.6) 5 (1.9) 17 (6.4) 19 (7.2) 45 (17.1) 10 (3.8) 37 (14.0)	258 (47.5) 6 (1.1) 33 (6.1) 40 (7.4) 93 (17.1) 39 (7.2) 73 (13.4)
DWI lesion volume (mL), median IQR	5.6 (2.3–16.6)	2.9 (0.95–11.9)	4.2 (1.5–13.8)
Thrombolysis, n (%)	60 (21.5)	73 (27.4)	133 (24.4)

### 4.2 LNM results of acute neurological deficits

**Fig. 1:**
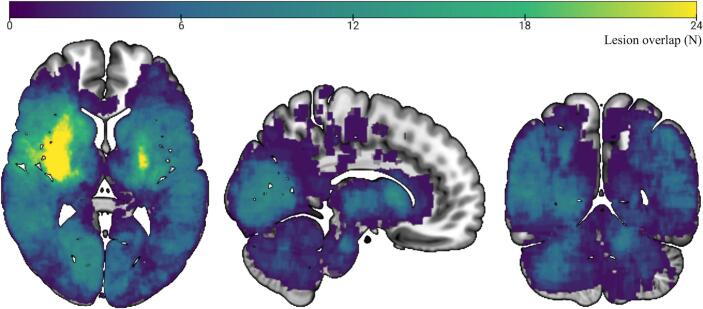
**Heatmap of Lesion Overlap Within The Total Patient Cohort**. The heatmaps illustrate the spatial distribution of lesions superimposed on a 100 μm Edlow background in MNI space. The colour bar indicates the number of lesions overlapping at the voxel level, with yellow representing the highest overlap and blue the lowest. The map highlights the maximum overlap of 24 lesions, providing a visual representation of lesion clustering and their spatial relationship within the entire study cohort, irrespective of NIHSS scores (N=545).

**Fig. 2:**
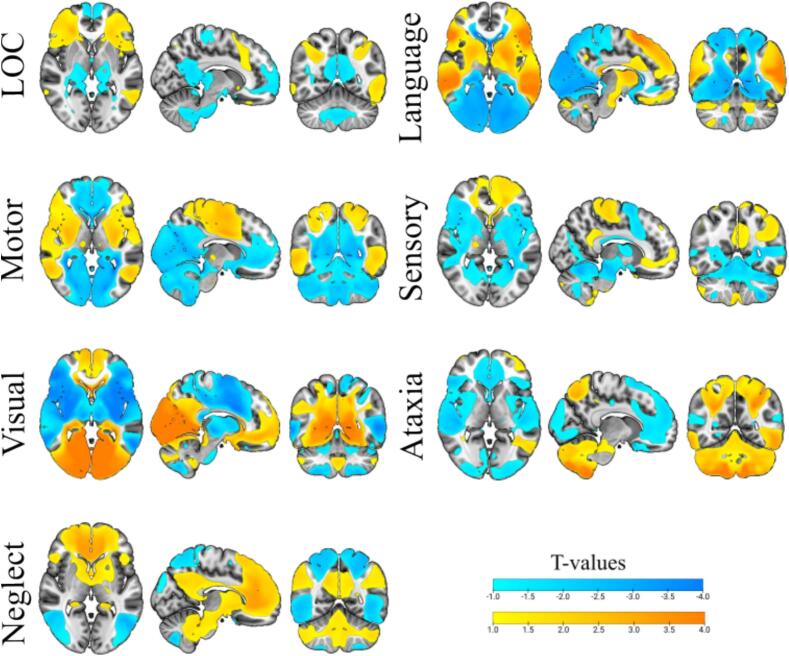
**NIHSS-derived Symptom Networks.** 2D surface maps of the brain highlighting regions functionally connected to lesion sites in patients who developed selected deficits after stroke; loss of consciousness (LOC), language deficit, motor deficit, sensory deficit, visual deficit, and presence of ataxia and neglect. The map display T-values to facilitate visualization. Each analysis was performed on the total sample of N = 545, with the number of patients classified as having a specific deficit varying by domain.

A heatmap illustrating the distribution of all included lesions in the pooled population is shown in Fig. 1**.** Lesion distribution stratified by the presence of specific neurological deficits is presented in **Supplementary Fig. S1.** All LNM analyses were performed based on binary scores within each of the seven symptom categories described in Table 1 and assessed in the pooled cohort of 565 patients**.** NIHSS-derived symptom networks for each of the seven symptom categories are displayed in Fig. 2**.** The LOC network (pFWE 0.00–0.05) involved bilateral regions across the frontal, parietal, and temporal lobes, including the frontal pole, inferior and middle frontal gyri, opercular cortices, insula, cingulate gyrus, and precuneus cortex. Subcortical structures such as the putamen, caudate, nucleus accumbens, pallidum, hippocampus, and amygdala were also implicated, as well as selected cerebellar regions (Crus I and VI). The language network (pFWE 0.00–0.05) showed a left-dominant network involving the superior and middle frontal gyri, precentral and paracingulate gyri, and subcortical structures including the pallidum, putamen, caudate, and thalamus. The motor network (pFWE 0.00–0.04) primarily involved bilateral regions across the frontal and parietal lobes, including the precentral and postcentral gyri, the supramarginal gyrus, angular gyrus, and the parietal operculum cortex. The sensory network (pFWE 0.00–0.05) involved bilateral regions, also including the postcentral and precentral gyri, with more frontal pole involvement. Subcortical structures included the thalamus, putamen, caudate, and the brainstem. The visual network (pFWE 0.00–0.04) showed strong connections within the occipital lobe, particularly involving the occipital pole, intracalcarine cortex, and lateral occipital cortex (superior and inferior divisions), as well as the cuneal cortex, supracalcarine cortex, and occipital fusiform gyrus. The ataxia network (pFWE 0.00–0.04) demonstrated strong connectivity with extensive cerebellar regions, including Crus I and II, lobules VI, VIIIa, VIIIb, IX, and X, as well as the vermis (VIIIa, VIIIb, IX, VIIb, and Crus II). Connections were more pronounced in the right hemisphere. Additionally, strong connectivity was observed with the brainstem, as well as the posterior division of the temporal fusiform cortex. The neglect network (pFWE 0.00–0.04) exhibited connectivity to parietal areas, including the superior parietal lobule, angular gyrus, parietal operculum cortex, and supramarginal gyrus. Additionally, it demonstrated connectivity to frontal regions, such as the superior and middle frontal gyri and the frontal pole. The network also involved extensive subcortical structures, including the thalamus and basal ganglia (caudate, putamen, and pallidum), as well as notable connectivity to the cerebellum, encompassing Crus I and II, lobules VI, VIIIa, VIIIb, IX, and X, along with their corresponding vermis regions.

A detailed summary of the anatomical regions identified within the respective symptom networks based on the Harvard-Oxford Cortical and Subcortical Structural Atlases (Desikan, 2006), and FNIRT-normalized cerebellar atlas (Diedrichsen et al., 2009) can be found in **Supplementary Table S1**.

### 4.3 Network damage scores

**Fig. 3:**
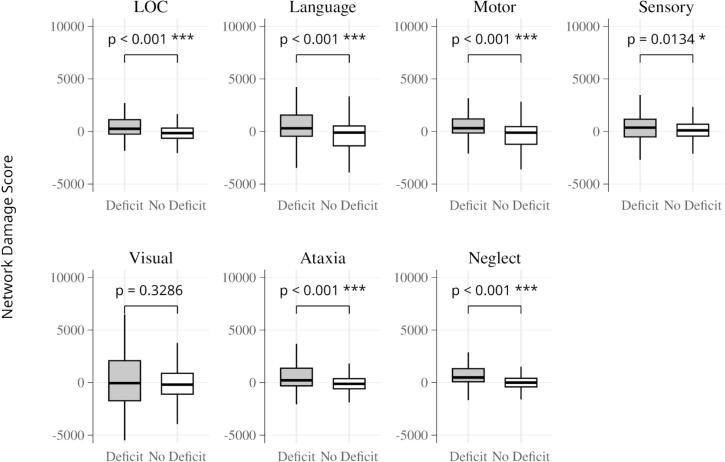
**Network Damage Scores by Symptom Category.** Boxplots showing network damage scores for patients with and without post-stroke deficits across seven symptom domains: loss of consciousness (LOC), language, motor, sensory, visual, ataxia, and neglect. Patients with deficits showed higher network damage scores in all categories compared to those without deficits, with significant differences in LOC, language, motor, sensory, ataxia, and neglect (p = 0.013–<0.001, Mann–Whitney *U* test), while the visual domain was not significant.

Network damage scores for each symptom category were calculated for every patient by identifying the intersection of their individual lesion with the corresponding NIHSS-derived symptom network derived from the pooled cohort analysis. For all seven symptom categories, we then compared network damage scores between patients with and without the corresponding deficit. For all seven symptom categories, the respective network damage scores differed statistically significant between patients with and without the corresponding symptom, as determined by the Mann-Whitney *U* test (Fig. 3). Effect sizes (Cohen’s d) ranged from small to moderate across domains. For each symptom category, the number of patients with the corresponding deficit (out of a total of 545 patients) and the associated effect size were as follows: LOC (d = 0.36, 73 patients with deficit), Language (d = 0.32, 290 patients with deficit), Motor (d = 0.42, 355 patients with deficit), Sensory (d = 0.21, 165 patients with deficit), Visual (d = 0.20, 121 patients with deficit), Ataxia (d = 0.50, 99 patients with deficit), and Neglect (d = 0.61, 45 patients with deficit).

### 4.4 Factors associated with functional recovery

We ran two ordinal regression models for functional outcome assessed via mRS. In Model 1, we included the following clinical variables: age, NIHSS score on admission, lesion volume, and NIHSS sub-scores for the seven pre-defined categories of acute neurological deficits (each treated as a continuous variable). In the multivariable analysis of Model 1, age (OR: 1.02, 95 % CI: 1.01–1.03, p < 0.001), LOC (OR: 2.1, 95 % CI: 1.3–3.5, p = 0.002), motor deficit (OR: 1.7, 95 % CI: 1.2–2.4, p = 0.002), sensory deficit (OR: 1.6, 95 % CI: 1.1–2.2, p = 0.003), and visual deficit (OR: 1.9, 95 % CI: 1.3–2.8, p = 0.001) were independently associated with functional outcome.

In Model 2, when NIHSS sub-scores were replaced with network damage scores, none of the network damage scores showed a significant association with mRS. In the multivariable model, only age (OR: 1.02, 95 % CI: 1.01–1.03, p < 0.001) remained significantly associated with functional outcome. Lesion volume (OR: 0.92, 95 % CI: 0.84–1.00, p = 0.100) and all network damage scores were not significant predictors (Table 3). Model comparison showed that Model 1 (clinical features only) fit better: it had a higher pseudo R^2^ (0.0468 vs 0.0159) and lower AIC (1730.598 vs 1769.222) and BIC (1799.411 vs 1833.707), indicating superior performance. Results were similar when analyzing each cohort separately (**Supplementary Table S2** for mRS assessed at 3 months within 1000Plus cohort and **Supplementary Table S3** for mRS assessed at one year within PROSCIS cohort). Predicted versus observed plots (**Supplementary Fig. S2**) showed that Model 1 predictions aligned more closely with functional outcomes, whereas Model 2 predictions clustered around lower values and did not capture higher disability levels.

**Table 3:** **Ordinal Logistic Regression Models for mRS assessed at 3-months and 1-year Post-Stroke.** Model 1 includes only clinical variables including presence of neurological deficits derived from composite NIHSS sub-scores, while Model 2 includes network damage scores based on NIHSS-derived symptom networks resulting from LNM analysis. For both models, the sample size was N = 545.

**Model 1 − mRS (ordinal)**	**Univariable model**		**Multivariable model**	
	**OR (95 % CI)**	**p-value**	**OR (95 % CI)**	**p-value**
Age in years	1.02 (1.0–1.0)	*<0.001*	1.02 (1.01–1.03)	<0.001
NIHSS on admission	1.2 (1.1–1.2)	*<0.001*	–	–
Lesion volume at baseline (log transformed)	0.9 (0.8–1.0)	0.035	0.9 (0.8–1.0)	0.058
Level of consciousness (LOC)	3.3 (2.0–5.2)	*<0.001*	2.1 (1.3–3.5)	0.002
Language deficit	1.3 (1.0–1.8)	*0.035*	1.0 (0.7–1.4)	0.800
Motor deficit	2.0 (1.4–2.8)	*<0.001*	1.7 (1.2–2.4)	0.002
Sensory deficit	1.7 (1.2–2.3)	*0.001*	1.6 (1.1–2.2)	0.003
Visual deficit	2.2 (1.5–3.2)	*<0.001*	1.9 (1.3–2.8)	0.001
Ataxia	0.9 (0.6–1.3)	0.561	0.8 (0.5–1.2)	0.298
Neglect	2.7 (1.6–4.8)	*<0.001*	1.5 (0.8–2.7)	0.180
**Model 2 (ordinal mRS)**	**Univariable model**		**Multivariable model**	
	**OR (95 % CI)**	**p-value**	**OR (95 % CI)**	**p-value**
Age	−	*−*	1.02 (1.01–1.0)	*<0.001*
Lesion volume at baseline (log transformed)	−	*−*	0.92 (0.84–1.0)	0.100
LOC network damage score	0.98 (0.91–1.05)	0.617	0.94 (0.83–1.0)	0.414
Language network damage score	1.00 (0.97–1.02)	0.974	0.94 (0.85–1.0)	0.330
Motor network damage score	1.00 (0.97–1.03)	0.706	1.07 (0.87–1.3)	0.474
Sensory network damage score	0.96 (0.89–1.03)	0.345	0.94 (0.83–1.0)	0.374
Visual network damage score	0.99 (0.97–1.01)	0.788	0.99 (0.85–1.1)	0.991
Ataxia network damage score	0.98 (0.94–1.03)	0.613	1.02 (0.92–1.1)	0.621
Neglect network damage score	1.01 (0.93–1.09)	0.763	0.94 (0.92–1.3)	0.281

Since the NIHSS total at admission was excluded from Models 1 and 2 to avoid collinearity with NIHSS sub-scores and network damage scores, we ran a separate ordinal regression with only age, log-transformed lesion volume, and NIHSS total (all continuous). In this model, NIHSS on admission was independently associated with mRS (adjusted OR 1.19, 95 % CI 1.14–1.25, p < 0.001). Age was also significant (adjusted OR 1.02, 95 % CI 1.01–1.03, p = 0.002). Log-transformed baseline lesion volume had adjusted OR of 0.92 (95 % CI 0.84–1.00, p = 0.062).

### 4.5 Sensitivity analysis

Spatial similarity scores for each symptom category were computed for every patient by correlating the individual lesion connectivity map with the corresponding NIHSS-derived symptom network. For all seven symptom categories, we then compared similarity scores between patients with and without the corresponding deficit. For all six symptom categories, similarity scores differed significantly between patients with and without the corresponding deficit, namely for LOC, Language, Motor, Sensory, Visual, and Ataxia; Neglect did not reach statistical significance. Effect sizes (Cohen’s d) ranged from small to moderate across domains. For each symptom category, the number of patients with the corresponding deficit (out of a total of 545 patients) and the associated effect size were as follows: LOC (d = 0.44; 73 patients with deficit), Language (d = 0.43; 290), Motor (d = 0.42; 355), Sensory (d = 0.27; 165), Visual (d = 0.39; 121), Ataxia (d = 0.54; 99), and Neglect (d = 0.25; 45).

In a supplementary ordinal regression for mRS, substituting network damage scores with NIHSS-derived spatial similarity scores did not alter the findings; incorporating these scores did not improve prediction of functional outcome (pseudo-R^2^ = 0.0159; AIC = 1769.362; BIC = 1833.846) **(Supplementary Table S4)**.

## 5 Discussion

In this study, we used LNM to identify specific symptom networks associated with acute neurological deficits, as assessed by individual NIHSS component scores. The identified networks were consistent with prior literature, highlighting the reliability of LNM in identifying functional symptom networks (Zarifkar et al., 2023, Ding, 2023, Ktena et al., 2019, Bowren, 2022, Salvalaggio et al., 2020). Our findings underscore the specificity of these networks, as evidenced by differences in network damage scores between patients with and without the respective neurological deficits. However, the inclusion of NIHSS-derived symptom networks did not enhance predictive performance beyond what was achieved with age and NIHSS scores alone in ordinal regression analyses. This suggests that while symptom-specific networks provide insight into the neuroanatomical basis of neurological deficits caused by ischemic stroke, they may not translate to improved prognostication of long-term functional outcomes.

Patients included in the current study were drawn from two independent prospective stroke cohorts. Patients from the 1000Plus cohort had higher cerebrovascular risk profiles and more severe strokes compared to patients from the PROSCIS-B cohort (Table 2). This may be due to the advanced age in 1000Plus patients; PROSCIS-B also only included first-ever stroke patients and was therefore inherently younger. Involvement of the arterial territories was similar across cohorts and lesion coverage overall across the pooled cohort was high (Fig. 1). Overall, the pooled study cohort represents a mild to moderate stroke population comparable to cohorts described in the literature, in terms of patient demographics and stroke etiologies (Liman, 2013, Hotter, 2009, Adams, et al., 1993). Patients in the 1000Plus cohort had higher NIHSS scores despite smaller lesion volumes, which may be explained by a higher frequency of strategic infarcts involving eloquent brain regions. Furthermore, the older age and greater burden of comorbidities in this cohort could contribute to increased clinical severity even with smaller lesions.

The results of the LNM analyses provide a comprehensive characterization of the neural networks associated with acute neurological deficits in ischemic stroke patients (Fig. 2**; Supplementary Table S1)**. Each of the seven symptom networks showed distinct connectivity patterns consistent with the known functional anatomy of these deficits. For example, the LOC network involved bilateral frontal, parietal and temporal regions, as well as critical subcortical structures such as the putamen, caudate, and hippocampus, highlighting the distributed network underlying arousal and consciousness (Koch et al., 2016, Snider, 2020). Motor and sensory networks showed overlapping regions within the frontal and parietal lobes, such as the precentral and postcentral gyri, but with differences in subcortical and brainstem involvement, underscoring their complementary roles in motor execution and sensory processing (Jimenez-Marin, 2022, Dulyan, 2022). The visual and neglect networks were notable for their focus on occipital and parietal areas, respectively, with the visual network showing strong connections to the occipital pole and intracalcarine cortex, and the neglect network showing robust parietal and frontal connectivity, consistent with spatial attention systems (Moore et al., 2023). The ataxia network was characterized by extensive cerebellar involvement, particularly in Crus I and II, lobules VI, VIII, and IX, and the brainstem, highlighting the critical role of the cerebellum in motor coordination. Interestingly, the language network identified in this study exhibited largely symmetrical connectivity patterns, involving the bilateral superior and middle frontal gyri, the precentral gyrus, and the thalamus. Previous studies, however, have reported left-dominant connectivity patterns in line with the known lateralization of language functions (Stockert, 2023, Rangus, 2024). The lack of clear lateralization in our NIHSS-derived language network may be partly explained by the higher proportion of right-hemispheric ischemic lesions in our cohort (Fig. 1), which is a common feature of prospective stroke studies, as patients with severe aphasia are often unable to provide informed consent.

The NIHSS-derived symptom networks identified in this study not only confirm the anatomical specificity of LNM-derived networks but also emphasize the distributed nature of brain connectivity underlying stroke-related deficits (Fig. 2). The comparison of network damage scores between patients based on the presence/absence of neurological deficits further shows a high specificity of the identified symptom networks **(**Fig. 3**)**.

Two ordinal regression models for functional recovery assessed at 3 months and 1-year post-stroke were analyzed. Model 1 included only clinical factors, including age, baseline lesion volume, and NIHSS total and component scores on admission. Age demonstrated a modest association with functional outcomes, consistent with its established role as a non-modifiable risk factor in stroke prognosis. Importantly, specific NIHSS sub-scores for LOC, motor, sensory, and visual deficits were also independently associated with mRS (Table 3**).** Importantly, in Model 2, where NIHSS clinical subscores were replaced with NIHSS-derived network damage scores, the predictive value of the NIHSS score in relation to LNM is lost. In fact, in Model 2, only age remained a robust predictor of outcome. This negative finding indicated that LNM does not provide prognostic information beyond what is already captured by clinical assessment alone. Direct model comparison via pseudo R-squared values, AIC, and BIC scores, as well as prediction–observation plots **(Supplementary Fig. 2)**, reinforces that network damage scores do not improve prognostic accuracy beyond established clinical variables. In sensitivity analysis, including NIHSS-derived spatial similarity scores, there was also no improvement in predictive accuracy of functional outcome. We consider this negative result an important contribution, as it highlights the limited clinical utility of LNM for outcome prediction, despite its value in mapping symptom topography. In other words, our study contributes to the ongoing debate on the clinical role of LNM, emphasizing that established clinical metrics such as the NIHSS remain more reliable for outcome prognostication in ischemic stroke.

In summary, the NIHSS sum score on admission remains a robust and reliable indicator of long-term functional recovery in ischemic stroke patients alongside advanced age. The prognostic value of incorporating symptom networks derived from specific acute neurological deficits, however, remains uncertain and warrants further investigation in larger, independent cohort studies (Jenkinson et al., 2012).

## 6 Strengths and limitations

There are several limitations in this study that deserve consideration. First and foremost, this is a retrospective analysis of prospectively collected data, which introduces inherent constraints in study design. Although functional outcomes were assessed using the mRS in both cohorts, the timing of these assessments differed (3 months in 1000Plus vs. 1 year in PROSCIS-B); previous studies have shown that up to 40 % of patients experience a shift in mRS scores between 90 days and 1 year (de Havenon, 2021). This potential variability could not be accounted for in the current study. Additionally, while the NIHSS score is a highly standardized tool administered by trained physicians in certified stroke units and has a high inter-rater reliability (Muir et al., 1996), it remains relatively rudimentary and may fail to capture subtle or complex neurological symptoms, such as coordination, gait impairment, distal motor function, vertigo, and cognition (Appelros and Terent, 2004). As a result, a detailed assessment of these stroke-induced symptoms and their potential impact on functional recovery may have been overlooked in the current study. Furthermore, by design, the individual NIHSS item scores are not equally weighted to different categories of neurological deficits. For instance, while motor and sensory deficits contribute up to 21 points to the total score, neglect contributes a maximum of only 2 points. This discrepancy should be considered when interpreting LNM results for each symptom category (Lyden, 2017). Also, although clinical NIHSS-based assessments were included as continuous variables in the regression models, the NIHSS-derived symptom categories were binarized for the subsequent LNM analyses to create NIHSS-derived symptom networks. This approach was chosen to reflect clinically meaningful domains and to ensure sufficient sample sizes for group comparisons, but it inevitably reduces granularity and may obscure subtle distinctions in deficit severity in the LNM analyses. Lastly, analyses with normative data reflect an indirect measure of functional diaschisis, without accounting for individual differences; however, a study comparing the use of a normative connectome and patient-specific data showed no substantial differences when employing either approach (Wang et al., 2021 Jan). Notably, an important advantage of using normative connectomes is that it allows us to analyze lesion data acquired in a clinical setting in which functional MRI acquisition is not standard.

Despite these limitations, our study has notable strengths. Most importantly, it draws on a relatively large sample of 565 S patients, combining comprehensive data from two independent, prospectively enrolled cohorts of mild to moderate ischemic stroke with long-term follow-up. Both cohorts underwent uniform assessment of acute neurological deficit using the NIHSS and functional recovery using the mRS, performed by trained and certified physicians. In addition, the use of high-resolution imaging (3 T MRI) and 3D binary lesion masks ensures high-quality LNM results. Finally, the analysis of acute stroke imaging lesion data from a large, comprehensive stroke cohort is unique in that it allows assessment of acute lesion effects on functional connectivity using a normative connectome. This provides insight into the underlying neural correlates of common acute neurological deficits and helps us to better understand their role in stroke prognosis.

## 7 Conclusion

In conclusion, this study demonstrates that although LNM is a valuable spatial mapping tool allowing for the identification of symptom-specific brain networks associated with acute neurological deficits after ischemic stroke, the added predictive value for functional outcome remains a matter of debate. The NIHSS score remains a robust and reliable tool for predicting long-term functional outcomes, underscoring its clinical utility. Future research may explore whether refinements in methodology or larger, more diverse cohorts can reveal subtle prognostic contributions of network-based measures.

## Declaration of Generative AI and AI-assisted technologies in the writing process

During the preparation of this work the authors used ChatGPT in order to improve readability. After using ChatGPT, the authors reviewed and edited the content as needed and take full responsibility for the content of the publication.

## CRediT authorship contribution statement

**Aslı Akdeniz:** Writing – review & editing, Writing – original draft, Visualization, Software, Methodology, Formal analysis, Data curation, Conceptualization. **Ana Sofía Ríos:** Formal analysis, Methodology, Software. **Uchralt Temuulen:** Formal analysis. **Jochen B. Fiebach:** Supervision. **Kersten Villringer:** Supervision. **Huma Fatima Ali:** Formal analysis. **Ahmed Khalil:** Validation, Supervision. **Ulrike Grittner:** Writing – review & editing. **Thomas Liman:** Writing – review & editing, Resources. **Matthias Endres:** Writing – review & editing, Supervision, Resources. **Anna Kufner:** Writing – review & editing, Writing – original draft, Funding acquisition, Formal analysis, Data curation, Conceptualization.

## Declaration of Competing Interest

The authors declare that they have no known competing financial interests or personal relationships that could have appeared to influence the work reported in this paper.

## Data Availability

Data will be made available on request.
